# Laparoscopic Anatomy of the Coelomic Cavity Organs in Female Red-Eared Slider (*Trachemys scripta Elegans*)

**DOI:** 10.1155/vmi/4041679

**Published:** 2025-07-24

**Authors:** Sepehr Pedram, Omid Zehtabvar, Amir Rostami, Hesameddin Akbarein, Shayan Zand, Yasaman Rezvani, Mohammad Mahdi Sari, Zahra Mollaei

**Affiliations:** ^1^Department of Surgery and Radiology, Faculty of Veterinary Medicine, University of Tehran, Tehran, Iran; ^2^Anatomy Sector, Department of Basic Sciences, Faculty of Veterinary Medicine, University of Tehran, Tehran, Iran; ^3^Department of Internal Medicine, Faculty of Veterinary Medicine, University of Tehran, Tehran, Iran; ^4^Department of Food Hygiene and Quality Control, Faculty of Veterinary Medicine, University of Tehran, Tehran, Iran; ^5^Faculty of Veterinary Medicine, Science and Research Branch, Islamic Azad University, Tehran, Iran; ^6^Faculty of Veterinary Medicine, University of Tehran, Tehran, Iran

**Keywords:** anatomy, coelomic cavity, laparoscopy, red-eared slider, surgical approach

## Abstract

**Background:** Turtles are one of the oldest reptiles and have evolved about 200 million years ago. One of them is the red-eared slider (*Trachemys scripta*). These freshwater turtles are native to the southern United States and northern Mexico. However, they have become popular pets in Iran and many countries around the world. Due to being non-native, they are considered one of the main dangers for the destruction of turtles' species in Iran and other countries. Therefore, knowing the location of coelomic cavity organs can be of great help in performing surgeries such as neutering in this species of turtle to prevent over reproduction and to preserve other species from the possibility of extinction and destruction.

**Objective:** Due to the fact that a precise descriptive study of the anatomy of the coelomic structures in the red-eared slider has not been conducted so far, and considering the necessity of having accurate information about coelomic anatomy for performing various surgeries using laparoscopic techniques, this study was carried out to determine the laparoscopic anatomy of the coelomic organs in red-eared sliders.

**Methods:** The turtles were off feed for 24 h before surgery. Anesthesia was induced with ketamine and diazepam and continued using the inhaler method. After scrubbing the prefemoral region and restraining, the center of the prefemoral fossa was sheared. The laparoscopy rigid lens was transmitted through this cut into the coelomic cavity without insufflation. After the completion of exploratory surgery, the cutting was closed.

**Results:** The study of coelomic cavity organs was performed on each of 5 turtles using laparoscopic technique with only one cut in the specified regions. The location and position of the organs were well determined to be used in future studies.

**Conclusions:** The use of laparoscopic technique is a practical and reliable way to examine the organs of the coelomic cavity. Although this method requires factors such as the ability of the surgeon to use the device, it is a noninvasive technique that helps the surgeon in accessing the organs away from the cutting site.

## 1. Introduction

Turtles are members of the oldest reptiles and having evolved around 200 million years ago. One of their species, the red-eared slider (*Trachemys scripta*), is native to freshwater habitats in the southern United States and northern Mexico. Nevertheless, they have become popular as pets in Iran and many other countries worldwide [1]. A main part of their various body systems is located within the coelomic cavity, protected by the shell structure [1–3].

Due to the hard structure of the turtle's shell, access to their coelomic cavity is challenging for clinicians to perform various examinations. Therefore, for clinical evaluation of the organs in this region and even for collecting samples from the coelomic organs, one of the less invasive methods is the use of laparoscopic techniques.

Zehtabvar et al. [4, 5] conducted an anatomical study on both respiratory and nonrespiratory structures within the coelomic cavity of the European Pond Turtle and investigated the topography of these organs.

In 2007, Innis et al. [6] performed ovariectomy on 11 mature female turtles, including 6 red-eared sliders, using laparoscopic techniques. They inducted anesthesia using a combination of ketamine and medetomidine, performed a small incision for endoscopy to guide them to the ovaries, and successfully removed the ovaries. One of the turtles, which belonged to a different species, passed away 7 days later because of lipidosis in the liver, and the second turtle from the same species died by reason of severe bacterial granulomatosis due to retained eggs.

Also in 2013, Innis et al. [7] performed orchiectomy using laparoscopy. This surgical procedure was conducted on 27 male turtles, of which 25 were young to mature red-eared sliders. In 22 cases, testicular removal was accomplished with a unilateral incision, while in 5 cases, bilateral incisions were required. The study's conclusion was that laparoscopic techniques represent a less invasive method for performing testicular removal in turtles.

In 2010, a study was performed by Divers et al. [8] and to evaluate celioscopy as a method for obtaining liver and kidney biopsies in turtles. This study aimed to examine the coelomic organs and collect samples from freshwater turtles. In this study, celioscopy was performed on 22 freshwater turtles, with endoscopy entering their coelomic cavity from both the left and right sides of the body.

Given that a precise descriptive study of the coelomic anatomy of the red-eared slider has not been conducted so far and also the requirement for accurate information on coelomic anatomy for various surgeries using laparoscopic techniques, this study was carried out to determine the laparoscopic anatomy of the coelomic organs in this species.

The aim of this study is to investigate the feasibility of performing laparoscopy through the prefemoral region approach and to assess and identify the positioning of coelomic organs with laparoscopic techniques through this approach. This study is the final and completed phase of another study conducted by part of the same group on the red-eared slider turtle. One of the similar studies conducted on this subject by the authors of this article in 2017 is a pilot study of the red-eared slider turtle, in which only a topographic study was conducted and no morphometric studies were conducted [9].

## 2. Materials and Methods

### 2.1. Individuals

Ten mature red-eared slider turtles (*Trachemys scripta*) were transferred to the dissection room of the Faculty of Veterinary Medicine, University of Tehran. They were kept in suitable conditions for 1 week to acclimate to the environment and coincide with their natural conditions. During this period, whole Kilka fish (Black Sea sprat) carcasses and dry food were used for feeding the turtles [2].

This study was a DVM thesis and all experimental procedures were approved by the Faculty of Veterinary Medicine, University of Tehran Local Ethics Committee (Registration number: 3816).

For the identification and separation of males and females, the key identification features provided in the sources were utilized. In this species, the plastron in males is concave, while in females, it is flat or raised. The tails are often longer and thicker in males.

In addition to the mentioned ten turtles, five mature female turtles were used for gross anatomical and morphometric studies. The average weight and length of these fifteen study samples were 545 ± 2.3 g and 21.5 ± 0.7 cm, respectively.

### 2.2. Gross Anatomical Study

At first, it should be mentioned that for this part of the study, no turtles were euthanized; instead, red-eared slider specimens that had been euthanized for unrelated reasons to our study were used. As previously mentioned, five female turtle specimens were included in this part of the study. In coordination with veterinary clinics and hospitals in Tehran, these five turtles, which had either been delivered to them dead or had to be euthanized for some reason, were collected. Problems noted that were unrelated to the objectives of this study included the following: oral administration of pentobarbital (EUTHASOL Virbac AH, Inc, 39% pentobarbital, 5% phenytoin sodium; 100 mg/kg dose per pentobarbital concentration) was used to euthanize the turtles, and all of them were euthanized within 24 h [10].

Based on the information obtained from anatomical studies of other turtles, dissections and anatomical assessments were conducted. Incisions were made at the junction between the carapace and plastron on both sides using scissors, and with additional incisions using a scalpel, connections to soft tissues under the plastron were severed. Therefore, the coelomic cavity became accessible for anatomical studies. To prevent the movement of the sample during dissection due to the curving shell, a specialized dissecting table was used. Various anatomical structures were examined step by step and were extracted from the coelomic cavity. During dissection, air was entered into the respiratory system using a syringe inserted into the trachea to allow the positioning of different parts to be assessed when the lungs were inflated, and air was released from the apparatus when necessary.

### 2.3. Morphometric Study

In the specimens evaluated in the gross anatomical study, some morphometric parameters of visceral organs were measured. Statistical analyses were done by SPSS software Version 26.0. The descriptive statistics are described by the mean ± SEM. Data were analyzed by one-way ANOVA and Tukey post hoc test. A *p* value less than 0.05 was statistically considered significant. To select the parameters, the study by Melero et al. on veiled chameleons and panther chameleons was used [11].

### 2.4. Laparoscopic Anatomy Study

**Figure 1:**
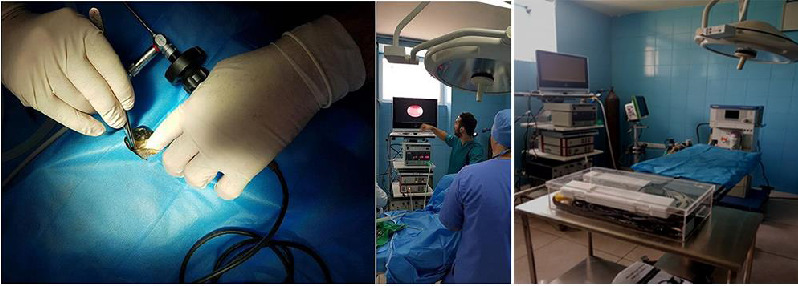
The procedures of performing a laparoscopic study of the coelomic cavity of a female red-eared turtle.

**Figure 2:**
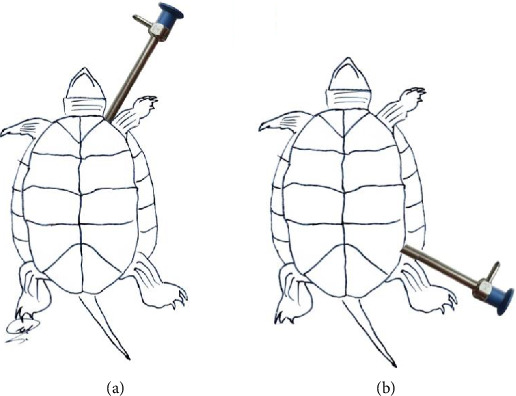
Two laparoscopic approaches used in this study. (a) left axillary and (b) left prefemoral.

It should be mentioned that all the tools and equipment used in this part of the study were made by the German company Karl Storz. After clinical examinations and standard preoperative tests, followed by a 24 h fasting period, the turtles were prepared for anesthesia. For preanesthesia, two drugs, ketamine (10 mg/kg) and medetomidine (0.2 mg/kg), were administered intramuscularly in the forelimbs. Subsequently, Isoflurane gas was delivered to the turtles through an appropriate mask into their respiratory passages. After the anesthetic procedures, the turtles were placed in a dorsal position on a heating pad, and one of the hindlimbs (preferably the left one) was extended for access to the target area (prefemoral region) and immobilized. This area, along with parts of the shell that could be in contact with the tools, was prepared in a standard aseptic manner, scrubbed with betadine and sterile gauze using circular scrubbing motions, followed by draping. In the center of the prefemoral region's skin on the left side, a 5-mm incision was made for the trocar camera insertion (Figures 1 and 2). Through the trocar placed in this hole, a rigid 5-mm lens with a 12-degree head angle entered the coelomic cavity. In addition, two 2 mm trocars were inserted, one next to the first trocar and the other in the prefemoral region on the right side, and two grasping instruments for mini laparoscopy were inserted into this cavity. The liver, stomach, intestines, heart, kidneys, lungs, bladder, and ovaries were visualized and examined. After exploratory laparoscopy, the aponeurosis was sutured with a simple interrupted suture using 3-0 Dexon and the skin was sutured with 3-0 Nylon. In the method of laparoscopy using the axillary approach (Figure 2), as in the previous method, the target area was scrubbed and draped for aseptic surgery. Then, a 5-mm incision was made in the skin of the left axillary region for the camera trocar insertion. Through this trocar placed in this hole, a 5-mm rigid lens with a similar angle to the previous approach was inserted into the coelomic cavity. In addition, two 2 mm trocars were inserted, one next to the first trocar and the other in the right axillary region. Using this approach, the esophagus, heart, and lungs were visualized and examined. After exploratory laparoscopy, the aponeurosis was sutured with a simple interrupted using 3-0 Dexon, and the skin was sutured with 3-0 Nylon. It should be noted that the left axillary and left prefemoral approaches were chosen based on other studies on turtles.

Laparoscopic equipment used: Processor (CCD) device model 5512 made by Richard Wolf Company, Germany, Cold light device model 5551 made by Richard Wolf Company, Insufflator or gas blower model 264,305 made by Störtz Company, Germany, Professional 23-inch flat monitor AOU, disposable laparoscopic trocar and Cannula (5 mm) (Figure 1). Figure 2 also shows the laparoscopic approaches.

## 3. Results

In this study, the location and positioning of internal organs within the coelomic cavity were examined using laparoscopic methods and compared with common anatomical study methods and then the approach to accessing each of these organs in laparoscopy was determined, and this is discussed in this section (Table 1). At first, review the results of the gross anatomical studies and assess the positioning of various organs within the coelomic cavity.

### 3.1. Gross Anatomical Study

**Figure 3:**
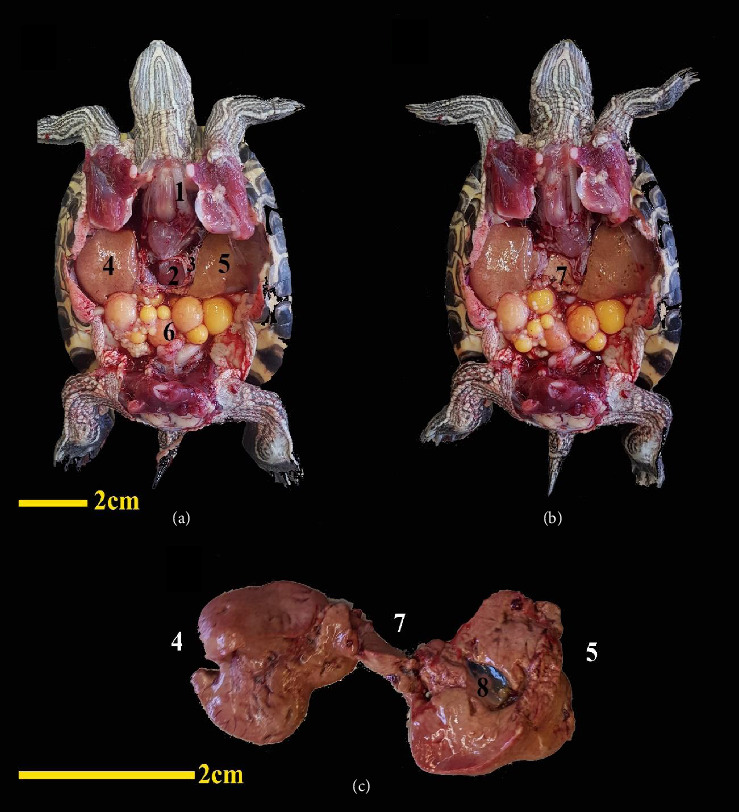
Ventral view of a female red-eared turtle. The plastron has been removed along with associated soft tissue (a-b); in image B, the heart has been removed and the middle lobe of the liver is visible. Dorsal view of the liver at higher magnification outside the coelomic cavity (c). (1) Trachea, (2) heart, (3) pericardium, (4) right liver lobe, (5) left liver lobe, (6) ovary, (7) middle liver lobe, and (8) gallbladder.

The first organ examined in the dissection was the heart. The heart was situated on the midline of the body and in the cranial half of the coelomic cavity within the pericardial cavity. This organ was separated from other parts of the coelomic cavity by a thin membrane. At the caudodorsal aspect of the heart, there was a connecting piece between the right and left lobes of the liver. Structurally, the heart was a muscular organ and had a relatively dark color (Figure 3).

The liver was one of the largest visceral organs, located ventrally to the surrounding visceral organs and deep in the shoulder girdle and peritoneum. The liver had a dark to reddish brown color. It had two main lobes, the right and left, with an intermediate lobe between them. The right lobe was larger, and the gallbladder was located adjacent to this part. The gallbladder was positioned near the dorsal surface of this lobe. An intermediate lobe or connecting lobe between the right and left lobes of the liver was at the dorsal surface of the heart, visible upon removing the heart. At the dorsal surface of the left lobe of the liver, the stomach was located (Figure 3). The bile duct emptied into the duodenum.

**Figure 4:**
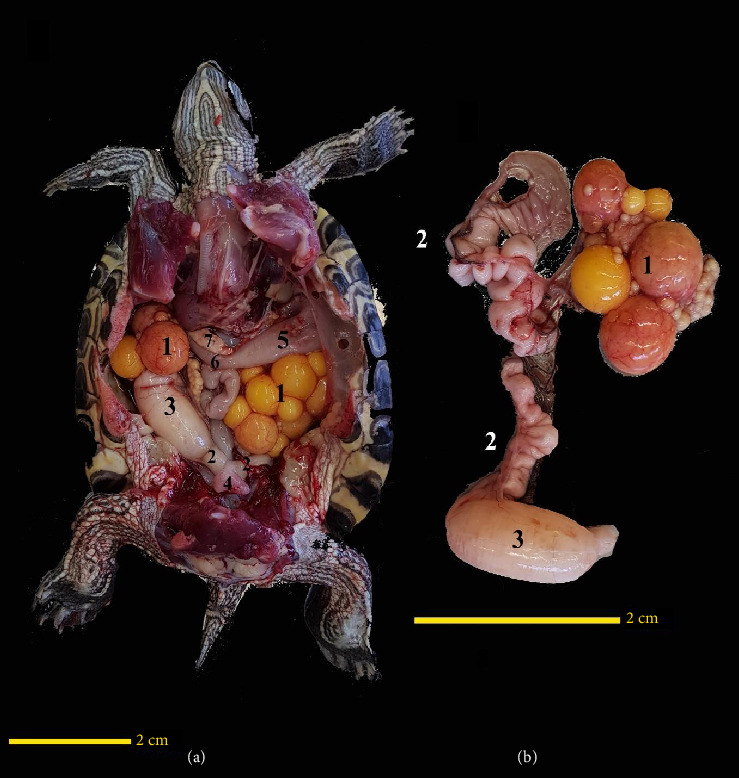
Ventral view of a female red-eared turtle: the plastron has been removed along with associated soft tissue, and the liver has also been removed (a) and ventral view of the right ovary and oviduct at higher magnification outside the coelomic cavity (b). (1) Ovary, (2) oviduct, (3) egg in the oviduct, (4) urinary bladder, (5) stomach, (6) duodenum, and (7) pancreas.

The ovaries were located ventrally to the kidneys. The oviducts were positioned ventrolaterally to the kidneys and cranially extended ventrolaterally to lungs. In one of the turtles dissected, a whole of eggs was found in the coelomic cavity, both on the left and right sides. Inside the right oviduct, one egg was in the final stages of development and entering the cloaca. The opposite side did not have a similar condition. It was notable that ovaries in this sample occupied the major part of the coelomic cavity (Figure 4). One important feature of the oviducts was their high degree of contortion, especially in the distal regions close to the cloaca (Figure 4).

**Figure 5:**
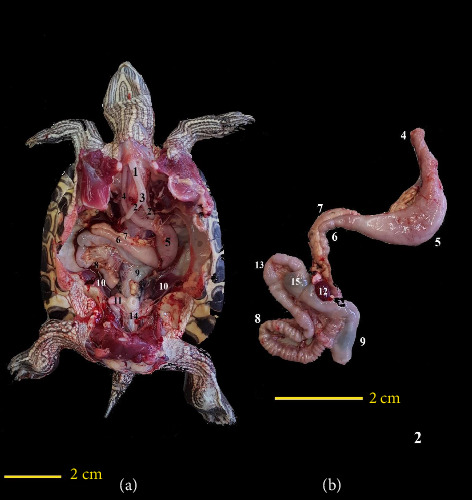
Ventral view of a female red-eared turtle: the plastron has been removed along with associated soft tissue, and in addition, the liver, ovaries, and oviducts on the right and left have been removed (a). Ventral view of the digestive tract from the esophagus to the cloaca at a higher magnification outside the coelomic cavity (b). In this image, the spleen has been displaced between adjacent parts of the intestines to be visible. (1) Trachea, (2) principal bronchus, (3) tracheal bifurcation, (4) esophagus, (5) stomach, (6) duodenum, (7) pancreas, (8) jejunum, (9) colon, (10) kidney, (11) urinary bladder, (12) spleen, (13) ileum, (14) cloaca, and (15) cecum.

The esophagus had a tubular and muscular structure. Its beginning extended along the midline of the neck and ended in spindle-like-shape stomach on the left side. The length of the esophagus varied depending on whether the head was extended out of the shell or retracted into it. When the neck was retracted inside the shell, the esophagus was positioned entirely behind the neck and deviated to the right (Figure 5).

The stomach was situated on the dorsal surface of the left liver lobe. It had a muscular structure, spindle-like-shape, a pink color, and superficial blood vessels that made it easily distinguishable (Figure 5).

After the stomach, the intestines were located on the right side of the body. The intestines had a tubular structure with varying diameters in different sections. They also had a pink color and fewer surface blood vessels than the stomach. The pancreas was adjacent to the duodenum and continued to the jejunum contortion part. Finally, the ileum was located, and this whole complex was located on the right side of the coelomic cavity. After the ileum was the cecum, which was continued to the colon, the colon was deviated from the right side to the midline and moved toward the cloaca; in fact, the colon had a transverse section and a descending section. The colon had a greater ratio to the small intestine. The wall of the colon was thinner and had a darker color structure due to the contents of this part.

The pancreas was attached to the distal surface of the liver and was adjacent to the proximal part of the duodenum. It was placed from the pylorus to the site of the common bile duct entrance. The pancreas was mostly pink with a slightly white appearance and had a smooth and thick texture. In fact, the pancreas was located in proximity to the lesser curvature of the pyloric part of the stomach and the cranial border of the proximal part of the duodenum (Figure 5).

The spleen was positioned near the posterior end of the pancreas, on the right side of the stomach. It had a rounded and smooth shape with a dark red color (Figure 5). It was relatively more dorsal compared with intestines and pancreas.

The bladder was located at the bottom of the coelomic cavity, and the urethra was opened at the bottom of the bladder and connected it to the urodeum from below (Figures 4 and 5).

The kidneys were present in pairs at the posterior of the coelomic cavity and caudal to the lungs. The kidneys were attached to the carapace on both sides of the vertebral column and were nearly oval in shape, with a reddish color, tending toward black (Figure 5).

The cloaca had a common structure for the digestive, urinary, and reproductive systems. It began a little before the pelvic cavity and had a terminal opening at the tail. The end of the colon entered the cloaca caudoventrally (Figure 5).

The trachea had a tubular structure and entered the coelomic cavity from the left side of the neck. The cartilaginous rings of the trachea were complete. The trachea splits into two branches on the left side of the coelomic cavity and tends to the midline and forms the bronchi. The bronchi moved toward the caudal and lateral and entered the lungs. This entrance occurred when the head and neck were extended outside the shell; when they were flexed and inside the shell, the trachea needed to move cranially to reach the lungs. The lungs had a spongy structure and were mostly pink in color, attached to the carapace on both sides of the vertebral column. The lungs were positioned at the dorsal surface of other visceral organs in the coelomic cavity. The ventral surface of the lungs that was adjacent to the visceral organ was covered by a thin septum (Figures 4 and 5). The lungs were separated from other organs by membranous tissue. This structure was thicker and more defined in the posterior part (postpulmonary septum).

### 3.2. Morphometric Study

Using one-way analysis of variance, a statistically significant difference in width between different lobes was seen (*p* < 0.001) (Table 2). With Tukey's supplementary test, a statistically significant difference was seen between the right and left lobes (*p* < 0.001) (Table 2). With Tukey's supplementary test, a statistically significant difference was seen between the right and middle lobes (*p*=0.001) (Table 2). With Tukey's supplementary test, a statistically significant difference was seen between the left and middle lobes (*p* < 0.001) (Table 2).

Using one-way analysis of variance, a statistically significant difference was seen in the length of the liver between different lobes (*p* < 0.001) (Table 2). Using Tukey's supplementary test, a statistically significant difference was seen between the right and left lobes (*p* < 0.001) (Table 2). Using Tukey's supplementary test, a statistically significant difference was seen between the right and middle lobes (*p* < 0.001) (Table 2). Using Tukey's supplementary test, a statistically significant difference was seen between the left and middle lobes (*p* < 0.001) (Table 2).

**Figure 6:**
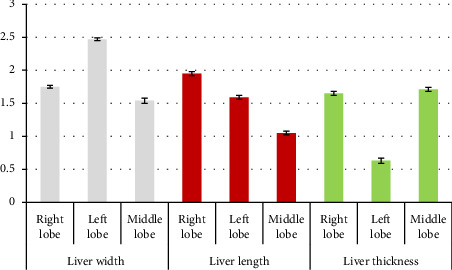
Morphometric parameters measured in three lobes of the liver of female red-eared slider liver (cm) mean ± SE.

Using one-way analysis of variance, a statistically significant difference in liver thickness between different lobes was seen (*p* < 0.001) (Table 2). Using Tukey's supplementary test, a statistically significant difference was seen between the right and left lobes (*p* < 0.001) (Table 2). Using Tukey's supplementary test, there was no significant statistical difference between the right and middle lobes (*p* = 0.411) (Table 2). Using Tukey's supplementary test, a statistically significant difference was seen between the left and middle lobes (*p* < 0.001) (Table 2). The morphometric parameters of different liver lobes are shown in Figure 6.

**Figure 7:**
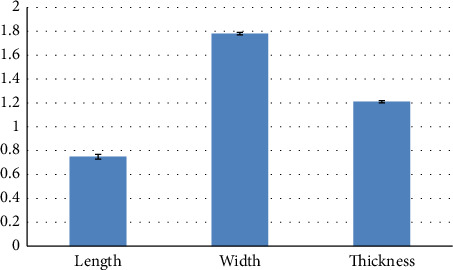
Morphometric parameters measured in the heart of female red-eared slider liver (cm), mean ± SE.

**Figure 8:**
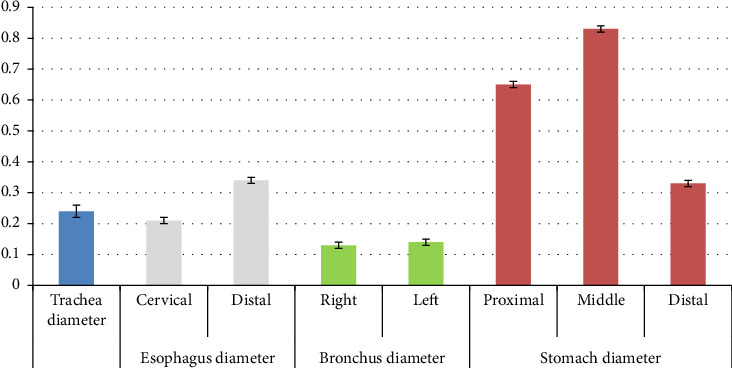
Morphometric parameters measured in the trachea, esophagus, bronchus, and stomach of the female red-eared slider liver (cm), mean ± SE (diameter).

**Figure 9:**
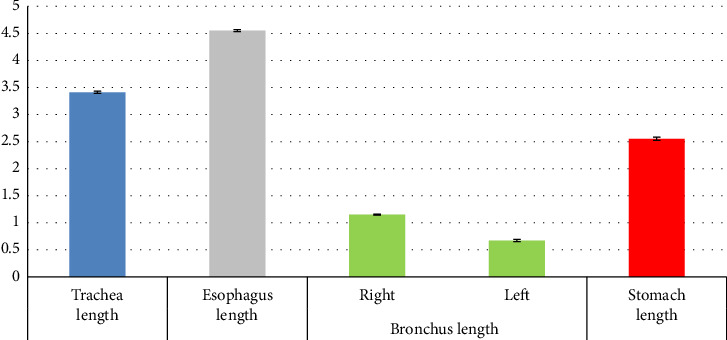
Morphometric parameters measured in the trachea, esophagus, bronchus, and stomach of the female red-eared slider liver (cm), mean ± SE (length).

Using the independent t-test, there was no statistically significant difference between the diameter of the trachea and the diameter of the cervical esophagus (*p*=0.228) (Table 2). Using the independent t test, a statistically significant difference was seen between the diameter of the trachea and the diameter of the distal part of the esophagus (*p*=0.002) (Table 3). Using the independent t-test, a statistically significant difference was seen between the diameter of the cervical esophagus and the diameter of the distal part of the esophagus (*p* < 0.001) (Table 3). Using the independent t-test, a statistically significant difference was seen between the diameter of the trachea and the diameter of the right bronchus (*p*=0.001) (Table 3). Using the independent t-test, a statistically significant difference was seen between the diameter of the trachea and the diameter of the left bronchus (*p*=0.001) (Table 3). Using the independent t-test, there was no statistically significant difference between the diameter of the right bronchus and the diameter of the left bronchus (*p*=0.347) (Table 3). Using the independent t-test, a statistically significant difference was seen between the length of the right bronchus and the length of the left bronchus (*p* < 0.001) (Table 3). Using the independent t-test, a statistically significant difference was seen between the diameter of the distal esophagus and the diameter of the proximal stomach (*p* < 0.001) (Table 3). Using the independent t-test, a statistically significant difference was seen between the diameter of the proximal stomach and the diameter of the middle stomach (*p* < 0.001) (Table 3). Using the independent t-test, a statistically significant difference was seen between the diameter of the proximal stomach and the diameter of the distal stomach (*p* < 0.001) (Table 3). Using the independent t-test, a statistically significant difference was seen between the diameter of the middle stomach and the diameter of the distal stomach (*p* < 0.001) (Table 3). The morphometric parameters of different lobes of the heart, trachea, esophagus, bronchi, and stomach are shown in Figures 6, 7, 8, and 9.

**Figure 10:**
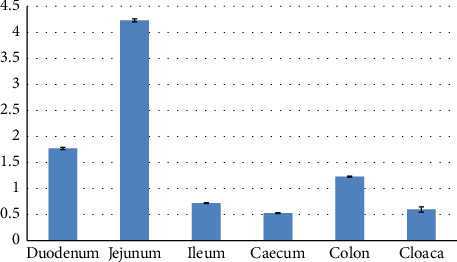
Morphometric parameters (lengths) measured in the duodenum, jejunum, ileum, cecum, colon, and cloaca of the female red-eared slider (cm), mean ± SE (length).

Using Tukey's supplementary test, a statistically significant difference was seen between the length of the duodenum and jejunum (*p* < 0.001). Using Tukey's supplementary test, a statistically significant difference was seen between the length of the duodenum and ileum (*p* < 0.001). Using Tukey's supplementary test, a statistically significant difference was seen between the length of the duodenum and cecum (*p* < 0.001). Using Tukey's supplementary test, a statistically significant difference was seen between the length of the duodenum and the colon (*p* < 0.001). Using Tukey's supplementary test, a statistically significant difference was seen between the length of the jejunum and ileum (*p* < 0.001). Using Tukey's supplementary test, a statistically significant difference was seen between the length of the jejunum and cecum (*p* < 0.001). Using Tukey's supplementary test, a statistically significant difference was seen between the length of the jejunum and colon (*p* < 0.001). Using Tukey's supplementary test, a statistically significant difference was seen between the length of the ileum and cecum (*p* < 0.001). Using Tukey's supplementary test, a statistically significant difference was seen between the length of the ileum and the colon (*p* < 0.001). Using Tukey's supplementary test, a statistically significant difference was seen between the length of the cecum and the colon (*p* < 0.001) (Table 4 and Figure 10).

**Figure 11:**
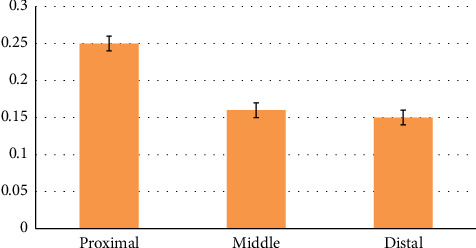
Morphometric parameters (diameters) measured in the proximal, middle, and distal part of duodenum of the female red-eared slider (cm), mean ± SE (length).

Using Tukey's supplementary test, a statistically significant difference was seen between the diameter of the proximal and middle parts of the duodenum (*p* < 0.001). Using Tukey's supplementary test, a statistically significant difference was seen between the diameter of the proximal and distal parts of the duodenum (*p* < 0.001). Using Tukey's supplementary test, there was no statistically significant difference between the diameter of the middle and distal parts of the duodenum (*p*=0.770) (Table 4 and Figure 11).

**Figure 12:**
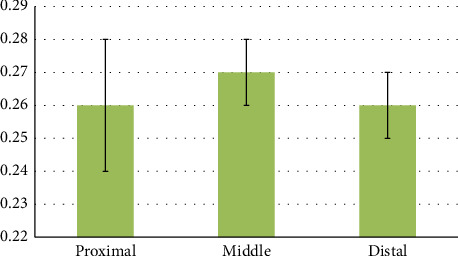
Morphometric parameters (diameters) measured in the proximal, middle, and distal part of the jejunum of the female red-eared slider (cm), mean ± SE (length).

**Figure 13:**
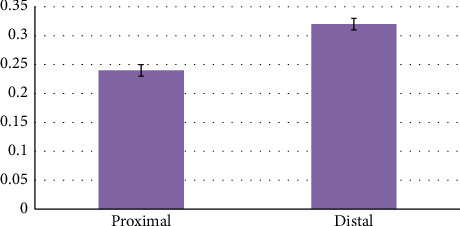
Morphometric parameters (diameters) measured in the proximal and distal part of the ileum of the female red-eared slider (cm), mean ± SE (length).

**Figure 14:**
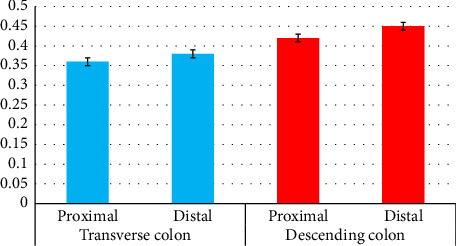
Morphometric parameters (diameters) measured in the proximal and distal part of the colon of the female red-eared slider (cm), mean ± SE (length).

Using the independent t-test, a statistically significant difference was seen between the diameter of the proximal and distal ileum (*p* < 0.001). Using the independent t-test, there was no statistically significant difference between the diameter of the proximal and distal transverse colon (*p*=0.232). Using the independent t-test, a statistically significant difference was seen between the diameter of the proximal and distal descending colon (*p*=0.027). Using the independent t-test, a statistically significant difference was seen between the diameters of the proximal duodenum and the distal stomach (*p* < 0.001). Using the independent t-test, a statistically significant difference was seen between the diameters of the proximal jejunum and the distal duodenum (*p*=0.001). Using the independent t-test, a statistically significant difference was seen between the diameters of the distal ileum and cecum (*p* < 0.001). Using the independent t-test, a statistically significant difference was seen between the diameters of the cecum and the proximal part of the transverse colon (*p* < 0.001). Using the independent t-test, a statistically significant difference was seen between the diameters of the distal part of the transverse colon and the proximal part of the descending colon (*p*=0.024). Using the independent t-test, there was no statistically significant difference between the diameter of the distal part of the descending colon and the diameter of the cloaca (*p*=0.184) (Table 4 and Figures 11, 12, 13, and 14).

### 3.3. Laparoscopic Anatomy Study: Left Prefemoral Approach

In Table 1, the organs observed in this approach are listed. Now, we will describe the details of what was observed for each of these organs.

**Figure 15:**
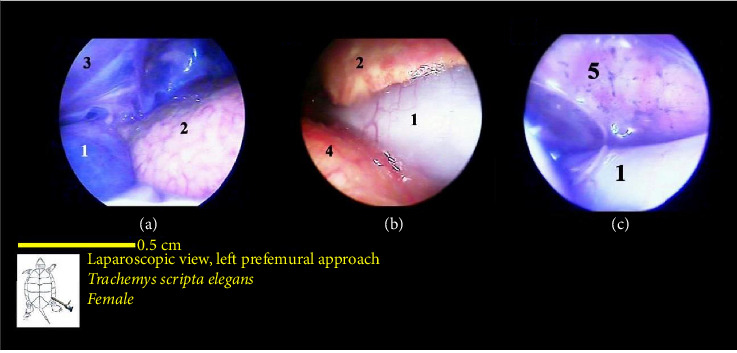
Laparoscopic view of the coelomic cavity of a female red-eared turtle, left perfemoral approach. (a) Left caudolateral view, (b) middle cranial view, and (c) right craniolateral view. (1) Stomach, (2) liver (left lobe), (3) postpulmonary septum, (4) liver (middle lobe), and (5) liver (right lobe).

The stomach was observed as a smooth structure covered in surface veins and was situated along with the left lobe of the liver on the ventral side of the left lung. These two structures were among the first organs observed upon entry through the prefemoral region. The stomach had a pink color and an abundance of surface veins (Figure 15).

**Figure 16:**
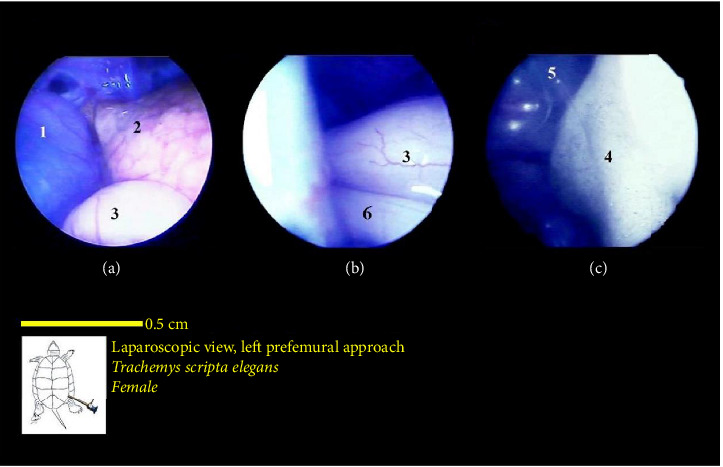
Laparoscopic view of the coelomic cavity of a female red-eared turtle, left perfemoral approach. (a) Left caudolateral view, (b) right caudolateral view, and (c) right lateroventral view. (1) Stomach, (2) liver (left lobe), (3) small intestine, (4) liver (right lobe), (5) right lung, and (6) large intestine.

**Figure 17:**
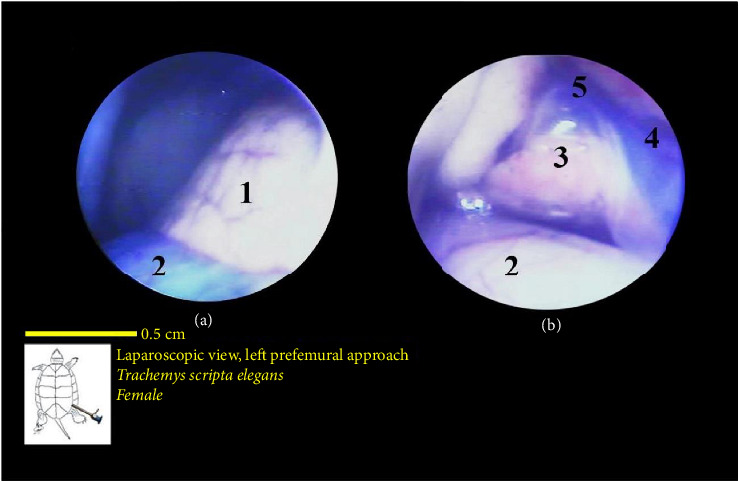
Laparoscopic view of the coelomic cavity of a female red-eared turtle, left perfemoral approach. (a) Left caudolateral view and (b) left lateroventral view. (1) Pancreas, (2) small intestine, (3) liver, (4) gall bladder, and (5) bile duct.

**Figure 18:**
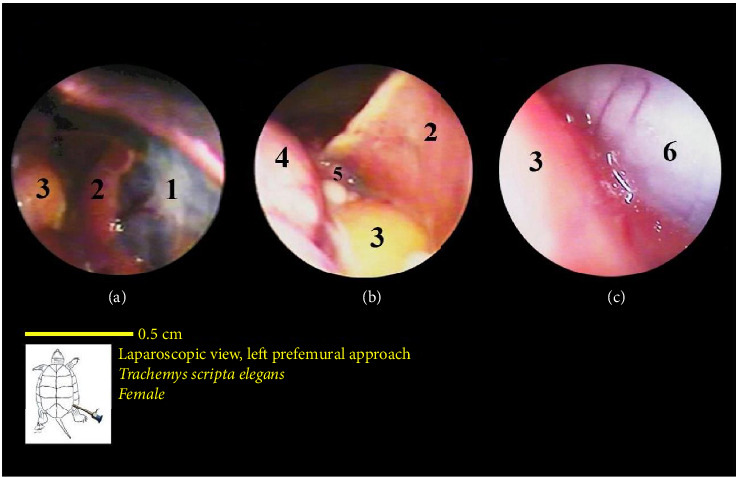
Laparoscopic view of the coelomic cavity of a female red-eared turtle, left perfemoral approach. (a) Left caudolateral view and (b) left lateroventral view. (1) Pericardium (heart), (2) liver, (3) ovary, (4) small intestine, (5) bile duct, and (6) colon.

The liver's location within the coelomic cavity was cranioventral. The liver featured two primary lobes, right and left, with an intermediate section (Figures 15 and 16). Between the two liver lobes, the heart was found caudally to the neck. Caudal to the liver, part of the small intestine was positioned. During laparoscopy, the intestines could be distinguished from the stomach, but the various segments of the small intestine were not easily discernible. The liver appeared in a dark red-brown color. It had a larger size compared with other organs. The gallbladder was positioned adjacent to the dorsal surface of the right lobe and followed by the bile duct (Figure 17). In the vicinity of the heart and the ova that extended in this area, the pericardium and heart were visible (Figure 18).

The intestines were situated caudal to the stomach and liver, and in the normal coelomic cavity, they were ventrally on the right side. During laparoscopy, they could be placed beneath the stomach or liver. The three principal parts of the small intestine, which include the duodenum, jejunum, and ileum, were not easily distinguishable from one another. Overall, the intestines had a tubular structure, were pink in color, and featured surface veins (Figure 16). It should be mentioned that these veins had a lower density compared with the surface veins of the stomach. The colon and cecum were not easily distinguishable from each other. They had a larger tubular structure than the small intestine. However, they had a darker color compared with the stomach and small intestine, and they also had fewer surface veins. The large intestine was located caudally in the coelomic cavity, and the different parts of the large intestine were not easily distinguishable from one another (Figure 16).

**Figure 19:**
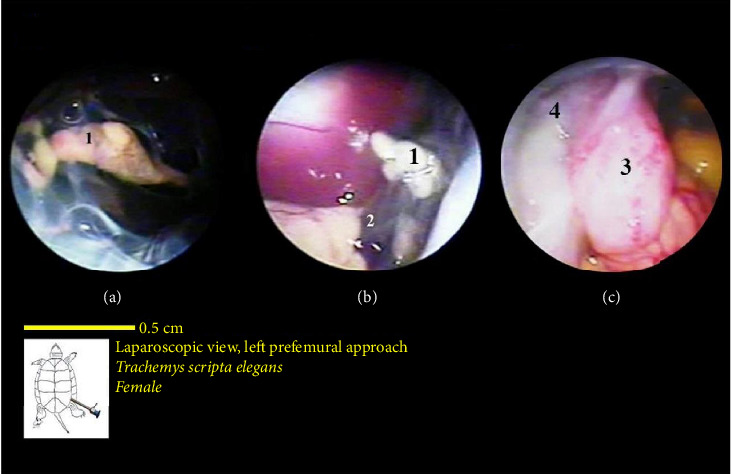
Laparoscopic view of the coelomic cavity of a female red-eared turtle, left perfemoral approach. (a) Left caudolateral view and (b) left lateroventral view. (1) Ovary, (2) kidney, (3) urinary bladder (completely empty), and (4) cloaca.

The lungs were observed as a pink, spongy tissue and were separated from the coelomic cavity by a thin wall. The kidneys were situated caudal to the lungs and were attached to the carapace. The kidneys had an oval-shaped structure with a reddish to brownish color and an irregular appearance (Figures 16 and 19).

**Figure 20:**
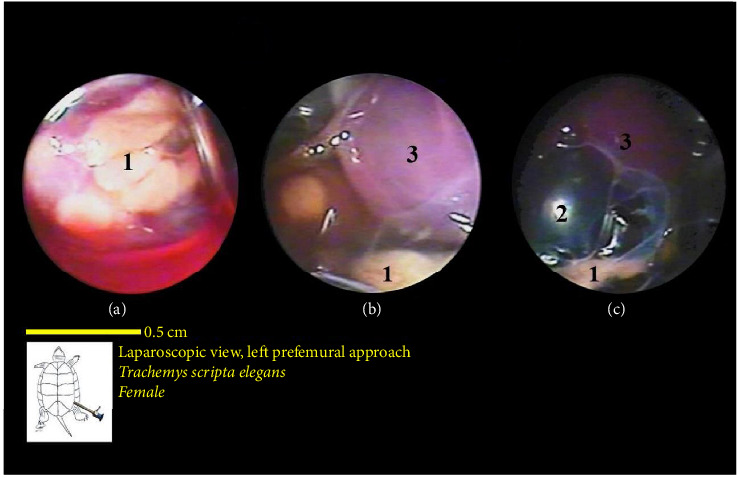
Laparoscopic view of the coelomic cavity of a female red-eared turtle, left perfemoral approach. (a) Left caudolateral view and (b) left lateroventral view. (1) Oviduct, (2) left kidney, and (3) urinary bladder (almost empty).

The bladder was located at the bottom of the coelomic cavity and had a thin wall. Its size varied significantly depending on whether it was full or empty. In most samples, the bladder was empty and occupied less space in the caudal part of the coelomic cavity (Figure 20).

The ovaries were positioned ventrally to the kidneys. The oviducts were situated dorsolaterally to the kidneys and extended cranially, ventrolaterally to the lungs. However, in one of the sexually active female turtles, the ova were well developed. Oviducts were broader, and these structures were observed in many caudal parts of the coelomic cavity. In addition, as an interesting finding, in one sample, the yolk structures and the egg were observed. In the vicinity of ova in various stages of development, near the body's midline, the colon was also visible.

### 3.4. Laparoscopic Anatomy Study: Left Axillary Approach

**Figure 21:**
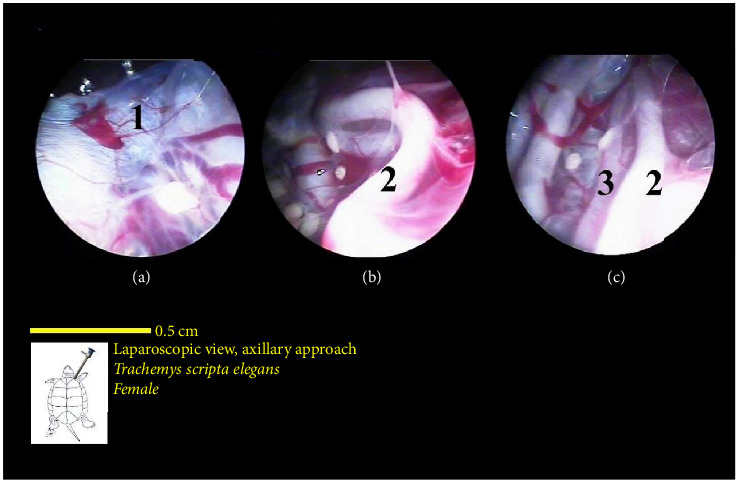
Laparoscopic view of the coelomic cavity of a female red-eared turtle, axillary approach. (a–c): Left craniolateral view. (1) Heart (pericardium), (2) esophagus in flexing neck, and (3) trachea.

The heart was located caudal to the neck between the two lobes of the liver on the midline of the body. The heart had a muscular structure with a beat, which reduced the rate of misdiagnosis. The heart was located inside the pericardium (Figure 21).

The trachea had a tubular structure and entered the coelomic cavity from the left side of the neck. The esophagus had a muscular structure, and its initial part was located on the midline of the neck and was very wide. The continuation of the esophagus in the neck extended state gradually inclined to the left and entered the stomach. But in the condition where the neck was contracted, the esophagus gradually inclined to the right, and after the end of the neck, it went to the left and moved a little to the front to reach the stomach (Figure 21).

In this study, the spleen and right and left bronchus were not observed in the laparoscopic method.

## 4. Discussion

In this study, the topography and determination of approach to the organs of the coelomic cavity in the female red-eared slider turtle were examined using laparoscopy and dissection, and differences and similarities with other turtles were observed, which will be mentioned in the following.

The left and right lobes of the liver are located on the medial surface of the bridge between the two parts of the shell and are protected by it. It should be noted that the color and different conditions of the liver are affected by environmental factors such as season and sexual conditions [12].

The gallbladder in the turtles used in the present study was located in the lower third of the coelomic cavity, closer to the floor of the plastron, while in the African star tortoise (*Centrochelys sulcata*), it is located in the middle third and below the lung [13].

It has been mentioned in many sources that the different parts of the intestine are not clearly distinguishable [12]. The way the esophagus is positioned and enters the stomach in the specimens examined in this study is similar to the European pond turtle (*Emys orbicularis*) and the Greek tortoise (*Testudo hermanni*), and no significant differences were observed [14].

The way the stomach is positioned in the red-eared slider is similar to the European pond turtle, and no significant differences were observed [14], but the way the stomach is positioned in these two is different from the Amazon river turtle (*Podocnemis unifilis*), in which the stomach is located almost transversely in the middle of the coelomic cavity, while in the other two species, it is located in the left half of the coelomic cavity. The pylorus of the stomach is located in the red-eared and European pond turtles, like the red-eared sea turtle, on the left side of the coelomic cavity near the midline of the body [15].

The whole arrangement of the small intestine portions is similar in all species of turtles. In the European pond turtle, the cecum appears to be a slightly enlarged portion of the beginning of the colon. Regarding the division of the colon parts, like the division done for the red-eared turtle, it is best to call the initial part, where the ileum empties and is located to the right of the midline, the proximal colon, and the terminal part, located on the midline of the body, the distal colon [13]. Other researchers have noted the differences in the structure of the digestive tracts of herbivorous and carnivorous turtles [16, 17].

In the red-eared and European pond turtles, the spleen is located to the right of the midline of the body, but studies conducted on the red-eared sea turtle have shown that this structure is located almost on the midline of the body and caudal to the heart, while the spleen was observed relatively further from the heart in our study [15].

Like other turtles, the kidneys are located retrocoelomically in the roof of the posterior part of the coelomic cavity, attached to the carapace [12].

In the specimens examined in this study, in some cases, the bladder was so full and expanded that it filled most of the posterior part of the coelomic cavity and displaced the intestines. References have mentioned the high elasticity of the bladder wall in turtles [4].

The very close proximity of the distal part of the large intestine to the kidneys, gonads, and their related ducts in the case of the passage of large digestive fragments or overgrown eggs in female turtles, considering the reduction of that part of the coelomic cavity due to the shape of the shell, seems to cause problems.

According to existing reports, the location of the trachea and bronchi in turtles is different. In the red-headed sea turtle, the trachea is located ventral and to the right of the esophagus [15]. In sea turtles, the tracheal bifurcation is slightly more posterior than in land turtles [18].

The lungs in the red-eared turtle are paired like other turtles; in addition, they are not located inside the pleural cavity and are attached to the carapace from above [19, 20]. In this study, a thin membrane was observed on the ventral surface of the lungs that was very transparent, and in other studies, the presence of a thin nonmuscular membrane that separates the upper part from the lower part of the coelomic cavity in turtles has been mentioned, and it has been called the postpulmonary wall. However, in studies conducted on the European pond turtle, in many cases, the structure of the lungs was observed between other viscera, especially the intestines, indicating that this membrane does not limit the lungs to prevent entry into the spaces between the organs and accompanies them in many cases [19].

According to the similar diameter of the trachea and cervical esophagus in the red-eared turtle, it should be noted that there is a possibility of misdiagnosis of these two structures during laparoscopy, especially since they are similar in location. Therefore, attention should be paid to the anatomical appearance of both.

Regarding the distal part of the esophagus and the initial part of the stomach, it should be noted that in terms of the anatomical appearance of the external surface of these two parts, they are similar, but the diameter of these two parts is different, and the initial part of the stomach has a larger diameter. Accordingly, this point should be taken into account when distinguishing the esophagus and the stomach.

Due to the similarity of the anatomical appearance of the outer wall of the different parts of the digestive tract, it is necessary to pay attention to the difference in the diameter of these parts and the topographic location of each one, and in this way, they can be separated from each other. One of the cases that may be mistakenly diagnosed with different parts of the digestive tract is the oviduct, and of course, its twisted appearance should be considered in this issue.

Based on the high diagnostic power and minimal invasiveness of laparoscopy, this method is one of the best diagnostic methods for pets. Considering that the internal structures of the turtle are located inside the protected space of the shell, performing examinations on them is accompanied by some limitations, so the use of the laparoscopic method on them is very helpful in diagnosing various problems. The use of the minimally invasive laparoscopic method can provide the possibility of viewing the organs inside the coelomic cavity in a desired way, and the entering of laparoscopic instruments can provide the conditions for accessing and displacing the organs inside the coelomic cavity in an appropriate way. In this study, the topography of various organs in the coelomic cavity of the red-eared turtle and the access to these organs were evaluated and providing a basis for further studies.

## Figures and Tables

**Table 1:** Organs that were observed in different approaches, in female red-eared slider.

Approach left axillary	Approach prefemoral left
Heart	Stomach
Trachea	Liver
Esophagus	Small intestine
—	Lunges
—	Kidneys
—	Oviducts
—	Urinary bladder
—	Bile duct
—	Gall bladder
—	Colon
—	Pancreas

*Note:* Organs not seen: spleen and bronchus.

**Table 2:** Morphometric parameters measured in three lobes of liver of the female red-eared slider liver (cm).

**Parameter**	**Liver width (right lobe)**	**Liver width (left lobe)**	**Liver width (middle lobe)**

Mean ± SE	1.75 ± 0.02	2.427 ± 0.02	1.54 ± 0.04

**Parameter**	**Liver length (right lobe)**	**Liver length (left lobe)**	**Liver length (middle lobe)**

Mean ± SE	1.95 ± 0.03	1.59 ± 0.03	1.05 ± 0.03

**Parameter**	**Liver thickness (right lobe)**	**Liver thickness (left lobe)**	**Liver thickness (middle lobe)**

Mean ± SE	1.65 ± 0.03	0.63 ± 0.04	1.71 ± 0.03

**Table 3:** Morphometric parameters^∗^ measured in the heart, trachea, esophagus, and stomach of the female red-eared slider (cm).

**Parameter**	**Heart length**	**Heart width**	**Heart thickness**

Mean ± SE	0.75 ± 0.02	1.78 ± 0.01	1.21 ± 0.01

**Parameter**	**Trachea length**	**Trachea diameter**	**Right bronchus length**

Mean ± SE	3.41 ± 0.02	0.24 ± 0.02	1.15 ± 0.01

**Parameter**	**Left bronchus length**	**Right bronchus diameter**	**Left bronchus diameter**

Mean ± SE	0.67 ± 0.02	0.13 ± 0.01	0.14 ± 0.01

**Parameter**	**Esophagus length**	**Esophagus (cervical) diameter**	**Esophagus (distal) diameter**

Mean ± SE	4.55 ± 0.02	0.21 ± 0.01	0.34 ± 0.01

**Parameter**	**Stomach (proximal) diameter**	**Stomach (middle) diameter**	**Stomach (distal) diameter**

Mean ± SE	0.65 ± 0.01	0.83 ± 0.01	0.33 ± 0.01

**Parameter**	**Stomach length**	—	—
Mean ± SE	2.55 ± 0.03	—	—

^∗^Regarding the parameters of width and diameter, the maximum amount of these parameters has been measured for each organ.

**Table 4:** Morphometric parameters^∗^ measured in three parts of the small and large intestine and cloaca of female red-eared slider (cm).

**Parameter**	**Duodenum length**	**Duodenum (proximal) diameter**	**Duodenum (middle) diameter**

Mean ± SD	1.77 ± 0.03	0.25 ± 0.01	0.16 ± 0.01

**Parameter**	**Duodenum (distal) diameter**	**Jejunum length**	**Jejunum (proximal) diameter**

Mean ± SD	0.15 ± 0.02	4.23 ± 0.07	0.26 ± 0.03

**Parameter**	**Jejunum (middle) diameter**	**Jejunum (distal) diameter**	**Ileum length**

Mean ± SD	0.27 ± 0.01	0.26 ± 0.03	0.72 ± 0.02

**Parameter**	**Ileum (proximal) diameter**	**Ileum (distal) diameter**	**Cecum length**

Mean ± SD	0.24 ± 0.01	0.32 ± 0.01	0.53 ± 0.03

**Parameter**	**Cecum diameter**	**Colon length**	**Colon (transverse part, proximal) diameter**

Mean ± SD	0.59 ± 0.03	1.23 ± 0.02	0.36 ± 0.02

**Parameter**	**Colon (transverse part, distal) diameter**	**Colon (descending part, proximal) diameter**	**Colon (descending part, distal) diameter**

Mean ± SD	0.38 ± 0.02	0.42 ± 0.02	0.45 ± 0.02

**Parameter**	**Cloaca length**	**Cloaca diameter**	—

Mean ± SD	0.60 ± 0.05	0.47 ± 0.01	—

^∗^Regarding the parameters of width and diameter, the maximum amount of these parameters has been measured for each organ.

## Data Availability

The data that support the findings of this study are available from the corresponding authors upon reasonable request.
